# Biophysical Insights into the Inhibitory Mechanism of Non-Nucleoside HIV-1 Reverse Transcriptase Inhibitors

**DOI:** 10.3390/biom3040889

**Published:** 2013-11-01

**Authors:** Grant Schauer, Sanford Leuba, Nicolas Sluis-Cremer

**Affiliations:** 1 Program in Molecular Biophysics and Structural Biology, Hillman Cancer Center, University of Pittsburgh, 5117 Centre Ave., Pittsburgh, PA 15213, USA; E-Mails: gds11@pitt.edu (G.S.); leuba@pitt.edu (S.L.); 2 Department of Cell Biology, Hillman Cancer Center, University of Pittsburgh, 5117 Centre Ave., Pittsburgh, PA 15213, USA; 3 Department of Medicine, Division of Infectious Diseases, 3550 Terrace St., Pittsburgh, PA 15261, USA

**Keywords:** HIV-1 reverse transcriptase, NNRTI, mechanism, biophysics, single-molecule

## Abstract

HIV-1 reverse transcriptase (RT) plays a central role in HIV infection. Current United States Federal Drug Administration (USFDA)-approved antiretroviral therapies can include one of five approved non-nucleoside RT inhibitors (NNRTIs), which are potent inhibitors of RT activity. Despite their crucial clinical role in treating and preventing HIV-1 infection, their mechanism of action remains elusive. In this review, we introduce RT and highlight major advances from experimental and computational biophysical experiments toward an understanding of RT function and the inhibitory mechanism(s) of NNRTIs.

## 1. Introduction

HIV-1 reverse transcriptase (RT) is an RNA- or DNA-dependent DNA polymerase and also contains ribonuclease H (RNase H) activity, thereby containing all the necessary enzymatic activity for the multistep conversion of HIV-1 single stranded RNA (ssRNA) into double stranded DNA (dsDNA) for subsequent integration into the human genome. RT thus remains the prime target for new antiretroviral therapies. Non-nucleoside RT inhibitors (NNRTIs) are potent inhibitors of reverse transcription, but despite having been successfully used in the clinic for over 15 years, a comprehensive explanation for their inhibitory mechanism(s) has remained elusive. 

## 2. Structure and Function of HIV-1 RT

### 2.1. Reverse Transcription

**Figure 1:**
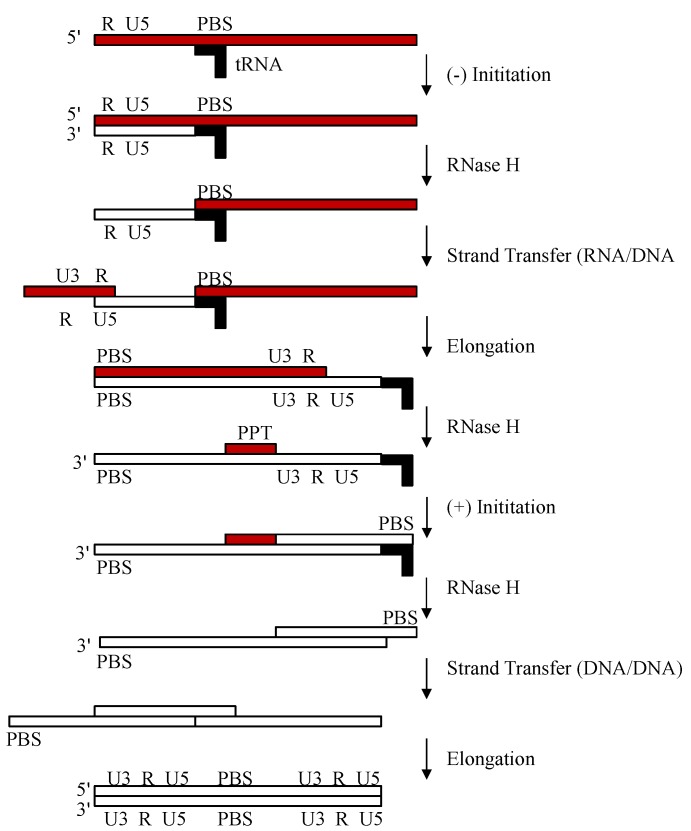
Reverse transcription. Schematic of the multistep process of the conversion of viral RNA (red) into integrase-competent dsDNA (bottom) for insertion into the human genome. PBS, primer binding site.

Reverse transcription [1,2] is initiated by RT at the 3'-end of cellular lysyl-tRNA^Lys,3^, which hybridizes to the primer binding site (PBS) on the HIV-1 genome. During initiation, RNA-primed RT elongates until the 5'-end of the HIV-1 RNA is reached, forming minus-strand strong-stop DNA (Figure 1). Employing the RNase H activity of RT, the remaining HIV-1 genomic RNA is cleaved to allow the nascently synthesized DNA to circularize and hybridize with the repeat sequence (R) at the 3'-end of the HIV-1 ssRNA. After this strand transfer, the nascent DNA strand is further elongated by RT. RT hydrolyzes the remaining RNA, but leaves behind a purine-rich sequence, named the polypurine tract (PPT), which subsequently serves as a primer for the initiation of second strand DNA synthesis. RT then elongates the PPT primer. The RNase H activity of RT removes all remaining RNA, including the transfer-RNA (tRNA) primer and the PPT. Strand transfer takes place by PBS sequence homology. DNA polymerization and strand-displacement followed by further DNA elongation results in an integrase-competent dsDNA, which is flanked by Long Terminal Repeat (LTR) sequences at both ends. 

The resulting dsDNA is then a substrate for integrase, which catalyzes the insertion of dsDNA into the human genome [2].

### 2.2. Structural and Biophysical Studies of RT

With the ability to efficiently catalyze DNA polymerization on both RNA/DNA and DNA/DNA duplexes and also possessing RNase H activity, RT is an astonishingly versatile enzyme, due in large part to its modular structure. RT is a 110 kD heterodimer composed of two subunits: p66 (560 amino acids-long) and p51 (440 amino acids-long) [3]. Both subunits are a product of proteolytic processing of a gag-pol polyprotein by HIV-1 protease. In p51, the RNase-H domain (residues 440–560) has been cleaved, resulting in a structure that shares secondary structural elements with p66. However, since the overall tertiary structure is spatially configured differently than p66, p51 largely plays a scaffolding role for p66, the only subunit in the RT heterodimer to possess polymerase and RNase H activity [4,5].

**Figure 2:**
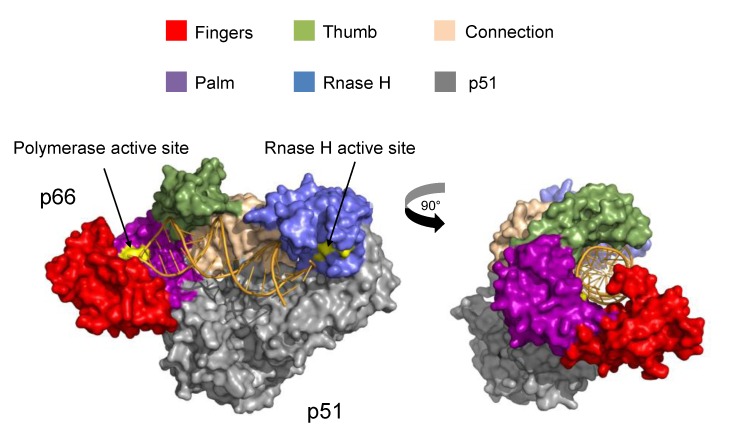
Structure of reverse transcriptase (RT). RT is pictured as a solvent-accessible surface area model (taken from PDB ID: 1RTD). The DNA/DNA template/primer is shown as a cartoon. The p51 subdomain is colored in grey, and the p66 subdomain is subdivided into thumb (green), fingers (red), palm (purple), connection (wheat) and RNase H (blue) domains. The polymerase and RNase H active site residues are colored in yellow.

X-ray crystallography, comprising the majority of biophysical studies on RT, has provided the community with an invaluable mechanistic understanding of RT. Beginning with the first structural elucidation of RT [5], there have since been hundreds of structures of RT solved (at the time of this writing, there were 245 structures of HIV-1 RT deposited in the Protein Database (PDB)). Additionally, a large body of mechanistic information complementary to the structural data has been gleaned from fluorescence-based kinetics studies. Like other DNA polymerases, RT is shaped like a right hand [5] (Figure 2).

Its subdomains are accordingly named: fingers (residues 1–85 and 118–155), thumb (residues 237–318), palm (residues 86–117 and 156–236) and connection (319–426) subdomains (see Figure 2). In the *apo* structure of RT [6], the thumb and finger domains are nearly in contact, and the thumb resides in the nucleic acid binding cleft; however, to accommodate template/primer (T/P) substrates in the nucleic acid binding cleft, the thumb must extend outward [6,7]. Pre-steady-state kinetic analysis showed that binding to the T/P and deoxynucleotide triphosphate (dNTP) substrates is a two-step process involving initial entry of the T/P into the nucleic acid binding cleft and a slow step dependent on the conformational change of the enzyme to accommodate cognate dNTP, which can increase the overall binding affinity to the T/P substrate [8]. The resultant binary complex contains a T/P substrate simultaneously spanned by polymerase and RNase H active sites, approximately 18 base pairs apart.

### 2.3. Conformational Dynamics of Reverse Transcription

RT clamps down on the incoming nucleotide with its fingers [9], specifically, via the Lys65, Arg72, Asp113 and Ala114 residues of the β3-β4 loop [10]. Structures of RT complexed with a DNA/DNA template/primer that has been dideoxy terminated at the 3'-end (to create a dead-end complex in the presence of cognate dNTP) identified the binding site of dNTP: Asp113, Tyr115, Phe116 and Gln151 [9]. In agreement from pre-steady-state kinetics data and based on data from other polymerases [11], it was proposed that finger clamping of dNTP is a rate-limiting prerequisite to catalysis [12]. Pre-steady-state kinetic analysis showed that dNTP incorporation rates depend upon the composition of the T/P substrate and suggested that RT can bind T/P substrates in productive or nonproductive complexes in terms of dNTP incorporation and that RT must undergo an isomerization step in order for the RT-T/P complex to be converted into a productive one [13]. The presence of this dead-end complex was later directly visualized with single-pair Förster resonance energy transfer (FRET) via multiparameter fluorescence detection (MFD) [14,15]. It has further been shown, through a combination of stopped-flow fluorescence experiments and a molecular dynamics technique used to extend the timescales of simulations, called directional milestoning [16], that the rate-limiting step of finger bending is on the order of milliseconds and that the conformational change itself governs dNTP specificity [17]. The bending of the finger clamp precisely positions dNTP for incorporation, which is coordinated with the positioning of the growing primer by the “primer grip”, composed of the β12-β13 hairpin [4]. Finally, formation of a nascent phosphodiester bond on a growing primer (*i.e.*, DNA polymerization) is coordinated by the catalytic triad in the palm subdomain (D110, D185, D186; part of the conserved YXDD motif, where Y and D stand for Tyrosine and Aspartate, respectively, and X stands for any amino acid), which is a conserved process amongst polymerases and is dependent upon two divalent metals [5]. The process of nucleotide addition ends in the release of pyrophosphate, which is accompanied by finger domain opening. In order for processive replication to continue, the T/P substrate needs to translocate one base pair with respect to RT, a process which is possibly linked to potential energy stored in the YMDD (Tyr-Met-Asp-Asp) loop, akin to a loaded springboard [18,19]. Consistent with this, atomic force microscopy (AFM) experiments demonstrated that finger clamping is likely responsible for generating the motor force necessary for RT translocation [20]. 

## 3. NNRTIs and Their Mechanism of Action

### 3.1. NNRTIs

**Figure 3:**
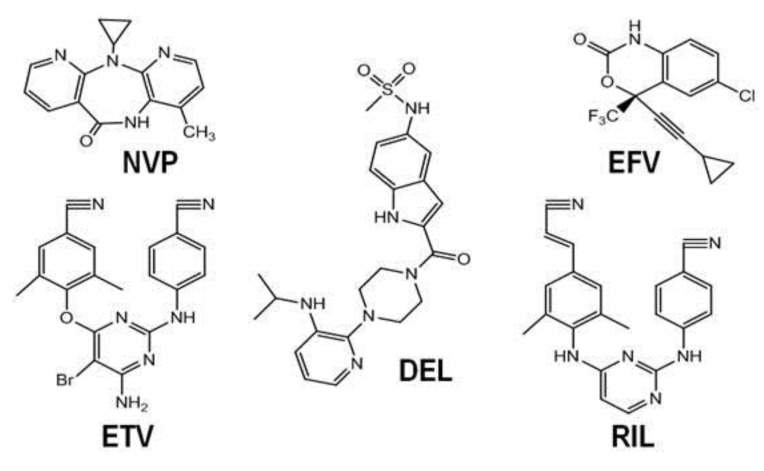
Chemical structures of USFDA-approved non-nucleoside RT inhibitors (NNRTIs).

NNRTIs are amphiphilic compounds that bind to a hydrophobic pocket in HIV-1 RT, called the NNRTI binding pocket (NNRTIBP), that is proximal to, but distinct from, the polymerase active site [21,22]. The NNRTIBP resides close to the p51-p66 interface and does not exist unless an NNRTI is bound [5,21]. NNRTIs typically bind with low nanomolar affinity and are potent inhibitors of reverse transcription [22]. In order of their approval from first to most recent, the five United States Federal Drug Adminstration (USFDA)-approved NNRTIs include nevirapine (NVP), delavirdine (DEL), efavirenz (EFV), etravirine (ETV) and rilpivirine (RIL) (Figure 3).

### 3.2. NNRTI Binding

Binding of NNRTI to RT is accompanied by rearrangements in the residues, Y181 and Y188, as well as in the primer grip motif, thereby forming the cavity of the NNRTIBP [5,6,21]. The fact that this pocket is not observed without the presence of NNRTIs has led some groups to conclude that an induced-fit mechanism [23] is responsible for NNRTI binding [24,25]; however, several other groups have demonstrated that conformational selection likely governs NNRTI binding [26,27,28,29]. Fourier transform ion cyclotron resonance mass spectrometry (FT-ICR MS) was originally used to show that p51 or p66 monomers undergo a population shift upon binding to EFV [28], and experiments monitoring EFV binding kinetics via tryptophan fluorescence were subsequently used to show that association of EFV to p51/p66 dimers was extremely slow, consistent with a conformational selection model [29]. Also consistent with this is data from nuclear magnetic resonance (NMR) experiments with RT that was site-specifically spin-labeled, which suggested that the primer grip was much more flexible than previously observed, presumably aiding in NNRTI binding [30]. The dynamic flexibility of the primer grip, comprising part of the NNRTIB, may provide evidence for conformational selection of NNRTIs. The likely important role of conformational selection has been further demonstrated using the Anisotropic Network Model [31] (ANM) combined with principle components analysis (PCA) methodology that both the unbound and NNRTI-bound conformation of RT intrinsically exist, but that NNRTI preferentially binds to conformations of RT within the structural ensemble, which are best predisposed toward NNRTI binding, shifting the population toward the NNRTI-bound form [27]. 

One obstacle to understanding the inhibitory mechanism of NNRTIs is that NNRTIs are practically insoluble in water at high concentrations (<10 mg/mL), such that traditional techniques for determining the equilibrium dissociation constant, K_d_, are made difficult. For instance, traditional K_d_ determination with isothermal titration calorimetry (ITC), the “gold standard” for measuring reaction thermodynamics, is made difficult, since it typically requires higher concentrations of protein (and, therefore, drug) than attainable for NNRTIs. Most studies therefore use polymerase activity assays to indirectly infer K_d_ for NNRTIs; however, the disadvantage to this technique is that it only measures dNTP incorporation, such that, e.g., if NNRTIs primarily had an effect on nonproductive binding, it would be difficult to determine an accurate K_d_. One alternative is surface plasmon resonance (SPR), which has been used to calculate K_d_ values for some NNRTIs [26,32,33]. Both SPR and ITC can be limited by extremely slow association and/or dissociation reactions, depending on the experiment [25,29]. One group circumvented the slow association of NNRTI to p51 monomers by extracting the K_d_ of EFV through the thermodynamic linkage between dimerization and NNRTI binding by combining kinetics from equilibrium dialysis, tryptophan fluorescence and native gel electrophoresis [29]. Another group recently surmounted the solubility barrier to ITC experiments by employing a technique using small, incremental injections of RT at regular intervals into the calorimeter (the Multiple Injection Method, or MIM [34]) containing fixed concentrations of NNRTI, demonstrating that, contrary to most reports, rather than acting on RT-DNA complexes, NNRTIs preferentially bind to free RT. This study showed that NNRTI-bound RT can then associate with DNA and form either a DNA-RT-NNRTI complex that exists in either a polymerase-competent incompetent configuration, both of which dNTP is unable to bind to [35].

### 3.3. Putative Mechanisms of Inhibition by NNRTIs

Interestingly, binding of NNRTIs does not appear to affect the ability of RT to bind incoming dNTPs [36,37]. Since the NNRTIBP is ~1 nm away from the polymerase active site and since binding of NNRTIs is noncompetitive with dNTP or template/primer binding [38], NNRTIs are considered classic allosteric inhibitors of HIV-1 RT DNA polymerization [21,39]. Despite their widespread use and remarkable efficacy, the precise mechanism(s) behind inhibition of reverse transcription by NNRTIs remains unclear [2,40,41,42], but proposed mechanisms have included restriction of thumb flexibility [5], distortion of the catalytic triad [43], primer grip repositioning [44], repositioning of the primer [45], enhancement of RNase H activity [46,47] and loosening of the thumb/finger clamp on template/primer substrates [48].

### 3.4. Molecular Arthritis

**Figure 4:**
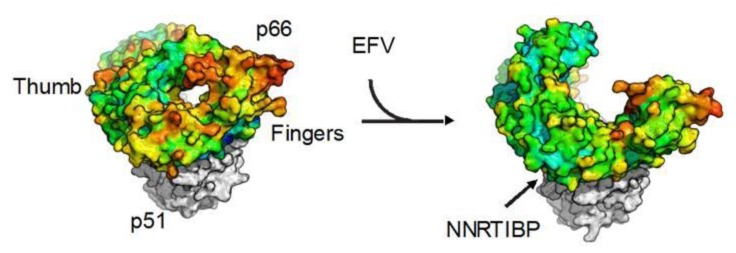
Molecular arthritis. The structures of *apo* wild-type (WT) RT (left; PDB ID: 1DLO) and WT RT bound to efavirenz (right; PDB ID: 1FK9). Structures are represented by solvent-accessible surface area. The p51 subunit is colored in grey, and the p66 subunit is colored according to crystallographic B-factors, with blue being the least mobile and red the most mobile residues. EFV, efavirenz; NNRTIBP, NNRTI binding pocket.

One persistent observation from the solved crystal structures of RT in the presence of NNRTIs has been the hyperextension of the thumb domain and a further opening of the finger domain accompanied by a marked decrease in flexibility, also known as “molecular arthritis [5]” (see Figure 4). Using hydrogen exchange mass spectrometry (HXMS), it was demonstrated that binding of EFV is allosterically coupled to regions of RT that are up to 6 nm away from the NRTIBP, primarily acting to stabilize the dynamics of these regions [39]. Elastic network models [31] and multicopy molecular dynamics simulations have shown that molecular arthritis appears to be closely related to the existence of a hinge region between the p51 and p66 subdomains [49,50]. Evaluating the slowest ANM modes, it was also shown that the presence of NNRTI changes the direction of finger and thumb motions, while the actual restriction of mobility was primarily seen in the presence of EFV, but not NVP [51]. Recently, however, one group using molecular dynamics simulations suggested that thumb mobility is not constrained as much as previously reported [52]. Notwithstanding, restriction of thumb flexibility has long been considered a key element in the inhibition mechanism [5], but whether molecular arthritis is directly and functionally tied to inhibition of polymerization is unclear. 

One possible effect of NNRTI-induced molecular arthritis is distortion of the catalytic triad [43]. In addition to the observation from crystal structures, repositioning of the catalytic triad into a polymerase incompetent configuration was directly observed in large, multicopy simulations of *apo* and NNRTI-bound RT [50]. Possibly linked to thumb hyperextension or simply to local rearrangements resulting from NNRTI binding, distortion of the precise constellation residues in the YXDD motif is presumed to be deleterious to polymerization activity. In a similar, but distinct, mechanism, it has also been postulated that NNRTI-induced molecular arthritis is responsible for repositioning the primer grip, eliminating the coordination of primer to the catalytic triad [44]. Furthermore, observation from crystal structures suggested that molecular arthritis is linked to the opening up of the RNase H domain, thereby providing access to degradation of DNA/RNA duplexes [53]. Consistent with this, EFV was shown to enhance RNase H activity [54].

### 3.5. Effect of NNRTIs on RT Dimerization

NNRTIs have been shown to enhance or stabilize the rate of RT heterodimerization, depending on the NNRTI studied, suggesting diversity in binding modes depending on chemical structure [55]. Perhaps counterintuitively, it has been demonstrated that some NNRTIs, such as EFV, act as potent chemical enhancers of HIV-1 RT heterodimerization [56,57]. To date, efavirenz was found to be the most potent enhancer of RT heterodimerization, whereas nevirapine has a weak effect, and delavirdine has no effect at all [56]. While some studies have demonstrated the effects of some potent NNRTIs, (e.g., efavirenz, dapivirine and etravirine) on the late stages of HIV replication [58,59], results from pre-steady-state kinetics experiments indicated that there does not appear to be a correlation between the impact of NNRTI-mediated enhancement of RT heterodimerization and the defects in RT polymerase function [60]. 

### 3.6. NNRTI Resistance Mutations

Due to the error-prone nature of RT, resistance mutations in and around the NNRTIBP arise during treatment regimens involving NNRTI therapy and are associated with virologic failure [61]. Because NNRTI resistance mutations are necessarily linked to RT function and to the mechanism of susceptibility of RT to NNRTIs, it is instructive to study them in order to better understand NNRTI mechanism [61]. It is generally understood that NNRTI resistance mutations can affect inhibitor binding by either altering one or more favorable interactions between the inhibitor and NNRTIBP (e.g., the Y181C mutation eliminates π-stacking interactions between this residue and the aromatic ring of the NNRTI [62]), by introducing steric barriers to NNRTI binding (e.g., G190E introduces a bulky side-chain, which may sterically interfere with NNRTI binding [63,64]) or by eliminating or altering the inter-residue network of the NNRTI-binding pocket, interfering with the ability of other residues in the pocket to envelop the NNRTI [21].

#### 3.6.1. K103N

Although a discussion of all resistance mutations is beyond the scope of this review, particularly in light of the accessibility of growing RT crystals for structural determination, resulting in an abundance of solved structures of RT with various resistance mutations, we will discuss K103N as a prime example of an NNRTI-resistance mutation, since it is the most commonly reported clinical mutation [65]. K103N confers high level resistance to a wide range of NNRTIs, notably efavirenz (EFV) and nevirapine (NVP) (approximately 20-fold reduction in susceptibility) [66]. Despite its devastating effects on susceptibility to first-generation NNRTIs, the mechanism of resistance by K103N remains unclear. Observations from the first structures of K103N RT [62,67] indicated that neither K103 nor the mutant N103 residue make any contacts with EFV or NVP (although K103 can H-bond with delavirdine (DEL)) [65]. It was initially proposed that the K103N mutation was responsible for adding an extra H-bond with Y188 in the NNRTIBP, stabilizing the *apo* form of RT [67]; however, the energy of an H-bond is relatively weak compared to that of NNRTI binding, and surface plasmon resonance (SPR) studies showed that K103N actually facilitates entry and exit into the NNRTIBP [38]. It was later inferred from a K103N/Y181C double mutant that the extra H-bond was possibly mediated by a Na^+^ ion and water molecules in the NNRTIBP [68]. Unlike first generation NNRTIs, the next generation diarylpyrimidine NNRTIs, etravirine (ETR) and rilpivirine (RIL), are effective against K103N RT [69,70,71,72,73]. At the moment, however, it is unclear what advantage these inhibitors have over first generation NNRTIs, although the crystal structures of K103N/Y181C RT and K103N/L101I RT suggest that the flexibility of ETR and RIL may allow the NNRTIBP to conform to these drugs, even in the presence of resistance mutations in the NNRTIBP [71]. It is also possible that the strengthened H-bond network conferred by K103N is broken by this new class of NNRTIs through a binding mode with extra favorable interactions [73]. Alternately, the diarylpyrimidine NNRTIs may be able to overcome K103N in a yet-to-be determined mechanism.

### 3.7. Structures of NNRTI-Bound RT-T/P Complexes

Until recently, there only existed structures of RT complexed with dsDNA template/primer (T/P) mimic substrates (mostly DNA/DNA, DNA/PPT) or structures of RT complexed with NNRTIs; however, there was no structure of a ternary RT-T/P-NNRTI complex. Recently, however, a structure of RT crosslinked to a DNA/DNA T/P substrate and bound to nevirapine (NVP) was elucidated [45], wherein the authors observed a distortion of the primer grip accompanied by thumb hyperextension and a 5.5 Å shift of the 3'-end primer away from the active site, dNTP binding site distortion and a reduction in RT-T/P contacts. By restricting the mobility of RT, it is possible that crosslinking of RT to the T/P substrate could lead to experimental artifacts. Soon after, structures of RT bound to several DNA/RNA T/P substrates in the presence of NVP and EFV were solved without the use of crosslinking agents [47]. The structure showed that NNRTIs pushed RT toward a degradative/RNase H-competent mode (as opposed to a polymerase competent mode) in the context of a hybrid substrate, which is consistent with the findings that EFV accelerates RNA degradation [54]. 

### 3.8. Insights into RT Dynamics from Time-Resolved Single-Molecule Experiments

Given the heterogeneity and complexity of the processes involved in reverse transcription and the suspected dynamic nature of RT-T/P interactions, there is great interest in observing single kinetic processes of reverse transcription. Due to the brief amount of time spent in the observation volume (~1 ms), single-molecule methods based on confocal fluorescence microscopy provide instantaneous, albeit detailed, structural information from FRET signals [14,15,74]. In contrast, time-resolved single-molecule techniques, relying on surface-tethering schemes, offer a glimpse into a broad range of dynamic processes for up to several minutes. One method to visualize RT dynamics on nucleic acid substrates is through direct visualization of the lengthening of flow-stretched, bead-tethered ssDNA [75] by RT as it completes primer extension [76]. Using this technique, different rates of primer extension and strand displacement were observed, and it was shown that RT dynamically switches between these modes, depending on the composition of the substrate [77]. Crucially, single-pair FRET experiments using total internal reflection fluorescence microscopy (TIRFM) [78] revealed that RT slides on nucleic acid substrates in a highly dynamic manner [48]. While cognate dNTP stabilized the polymerase competent configuration, NNRTI destabilized this configuration, enhancing the sliding dynamics of RT on the T/P. Remarkably, RT was shown to flip on template/primer substrates and can reside in different relative orientations based on the composition of the template/primer alone. It was further shown that RT bound T/P substrates in opposite orientations depending on whether it contains a DNA or RNA primer [77]. The relative orientation was a direct predictor of enzymatic activity; *i.e.*, when assayed for activity, the orientations were shown to be either polymerase-competent or RNase H-competent. When bound to a T/P with a PPT primer, RT could flip between these orientations, and the rate of flipping could be slowed by dNTP (directing RT toward a polymerase-competent configuration) or enhanced by NNRTIs (directing it toward an RNA-degradative orientation) [77]. Taken together, these results indicated that modulation of the grip on the T/P substrate itself by NNRTIs may alter RT-T/P dynamics, disfavoring the polymerase mode, suggesting that NNRTI-induced molecular arthritis affects the grip on the template/primer substrate. Intriguingly, it was recently demonstrated that the binding of the various dimeric isoforms of RT could be distinguished using a single-molecule fluorescence method, called Protein-Induced Fluorescence Enhancement (PIFE) [79], where the authors were able to monitor the binding of RT via the fluorescence intensity enhancement of a Cy3-labeled T/P resulting from the proximity of RT to Cy3 [80]. 

## 4. Conclusions and Future Directions

Combining results from many interdisciplinary biophysical experiments, a comprehensive picture of the mechanism(s) of action of NNRTIs is emerging, but is far from complete. Clearly, molecular arthritis plays a role in the inhibitory mechanism of NNRTI, but the exact link between modulation of structural dynamics as it relates to the attenuation of RT function remains unclear. Exciting new means of obtaining the crystal structures of T/P-bound RT in the presence of NNRTIs have emerged, and novel structures of T/P-bound, NNRTI-resistant RT mutants complexed to their relative NNRTIs are likely soon to follow. In X-ray crystallographic studies, crystal packing can lead to underestimation of flexibility, as shown for RT with Hydrogen-Deuterium Exchange Mass Spectrometry (HXMS) [81] and NMR [30], highlighting the necessity of using molecular dynamics simulations to probe the conformational dynamics of solved structures. Advances in sampling techniques for molecular simulations combined with the continually increasing computational power afforded by Moore’s Law [82] will help bring these structures to life, providing unprecedented knowledge of the conformational dynamics of reverse transcription and its inhibition by NNRTIs. Advances in the techniques used to quantify the thermodynamics of NNRTI binding may help in understanding the evolutionary rationale for certain NNRTI resistance mutations. Novel spin-label probes for NMR experiments will permit measurement of the dynamics of new regions of RT in the presence or absence of NNRTIs. Although traditional biophysical experiments have afforded crucial insight into the mechanisms of reverse transcription and its inhibition by NNRTIs, single-molecule biophysical experiments offer many strategic advantages over bulk experiments when it comes to understanding the kinetic mechanisms of a dynamic enzyme like RT. For instance, since traditional bulk fluorescence spectroscopy experiments represent an ensemble average of many unsynchronized kinetic processes occurring simultaneously (not all of which are related to the observable of interest), data from these techniques may be difficult to interpret [83]. Furthermore, crystallographic structures represent an ensemble average of structural states at equilibrium, but cannot provide information on the transitional intermediates between these states. Eliminating bulk averaging and enabling the visualization of rare events not associated with low-energy structures, single-molecule techniques will likely be increasingly used to resolve signals from individual RT complexes without the need for synchronization [83]. It will be interesting to see whether the techniques involving the multiplexed observation of flow-stretched DNA molecules will be put to use to probe NNRTI function. Completely new types of information have been made available from single-pair FRET, and these experiments are likely to continue. Although experiments cannot provide the detailed structural and/or orientation information, it is nonetheless an extensible technique that will likely see more use in the near future. Since PIFE does not require exogenous labeling of RT, it does not require structural perturbation and also may be more accessible to some laboratories than FRET. Furthermore, through observation of signals from T/P in the presence of unlabeled RT, single-molecule protein induced fluorescence enhancement (PIFE) experiments [80] allow the observation of physiologically relevant RT concentrations by providing a workaround to the “concentration problem” [83] seen in single-molecule FRET experiments, which are limited to the use of low nanomolar concentrations of labeled protein. Biophysical studies of RT structure, function and inhibition by NNRTI are all interrelated; e.g., the results from time-resolved single-molecule experiments must be informed by the structural data, the interpretation of which is, in turn, aided by new advances in computational simulation and biophysical techniques to measure protein dynamics. Combining these powerful methodologies, a great wave of enlightening structural information about HIV-1 RT is imminent, hopefully providing new strategies for rational drug design against this highly elusive target.
